# Does the functional polymorphism-1562C/T of MMP-9 gene influence brain disorders?

**DOI:** 10.3389/fncel.2023.1110967

**Published:** 2023-05-03

**Authors:** Sylwia Pabian-Jewuła, Marcin Rylski

**Affiliations:** ^1^Department of Translational Immunology and Experimental Intensive Care, Centre of Postgraduate Medical Education, Warsaw, Poland; ^2^Department of Radiology, Institute of Psychiatry and Neurology, Warsaw, Poland

**Keywords:** MMP-9, MMP-9-1562C/T polymorphism, rs3918242, brain diseases, brain

## Abstract

Metalloproteinase-9 (MMP-9) is one of the most strongly expressed matrix metalloproteinases (MMPs) in the brain. The MMP-9 activity in the brain is strictly regulated, and any disruptions in this regulation contribute to a development of many disorders of the nervous system including multiple sclerosis, brain strokes, neurodegenerative disorders, brain tumors, schizophrenia, or Guillain-Barré syndrome. This article discusses a relationship between development of the nervous system diseases and the functional single nucleotide polymorphism (SNP) at position -1562C/T within the MMP-9 gene. A pathogenic influence of MMP-9-1562C/T SNP was observed both in neurological and psychiatric disorders. The presence of the allele T often increases the activity of the MMP-9 gene promoter and consequently the expression of MMP-9 when compared to the allele C. This leads to a change in the likelihood of an occurrence of diseases and modifies the course of certain brain diseases in humans, as discussed below. The presented data indicates that the MMP-9-1562C/T functional polymorphism influences the course of many neuropsychiatric disorders in humans suggesting a significant pathological role of the MMP-9 metalloproteinase in pathologies of the human central nervous system.

## 1. Introduction

### 1.1. MMP-9 in brain physiology

Matrix metalloproteinases play an important role in the physiology of the nervous system, not only by degrading the extracellular matrix (ECM) but also by activation of biologically active substances in ECM, like for example, growth factors and their receptors. Metalloproteinase-9 is one of the most strongly expressed MMPs in the central nervous system (Fanjul-Fernández et al., 2010; Rivera et al., 2010).

The role of MMP-9 in neurobiology is intensely studied, however, our knowledge about it is still incomplete. MMP-9 is expressed in many areas of the brain, including the hippocampus (Aujla and Huntley, 2014), cerebral cortex (Bednarek et al., 2009), striatum (Dzwonek et al., 2004), and cerebellar cortex (Vaillant et al., 1999). At a cellular level, MMP-9 is mainly expressed in neurons (Szklarczyk et al., 2002), but also to a lesser extent it is found in astrocytes (Kinoshita et al., 2013) and microglia (Del Zoppo et al., 2007). The MMP-9 protein and its activities are identified mainly in the cell bodies of neurons, in dendrites, in excitatory synapses (Wilczynski et al., 2008; Gawlak et al., 2009), or thalamo-cortical synapses (Murase et al., 2019). MMP-9 plays a crucial role in the plasticity of dendritic spines (Pijet et al., 2019) and the formation and maintenance of perineuronal net integrity of the extracellular matrix (Murase et al., 2017). It also plays a crucial role in synaptic pruning (Bijata et al., 2017), transport of glutamate receptors (Michaluk et al., 2009), long-lasting synaptic enhancement (Vafadari et al., 2016), and myelination, synaptogenesis, and axon pathfinding (Reinhard et al., 2015). The last review also highlighted the role of the Rho family and GTPases as probably crucial players of MMP-9-controlled signaling in the remodeling of synapses during physiological and aberrant plasticity (Figiel et al., 2021). The MMP-9 expression is increased in the hippocampus during learning and memory formation (Vafadari et al., 2016; Beroun et al., 2019). However, the role of MMP-9 does not end with its involvement in the neurodevelopmental and neuroplastic processes. MMP-9 also plays a role in the modulation of cellular response to neuroinflammatory processes (Könnecke and Bechmann, 2013; Hannocks et al., 2019). Also, possible is the role of MMP-9 in arteriovenous growth and invasiveness (Panda et al., 2020).

MMP-9 expression is strictly regulated during a body’s development, with high levels at early developmental points, and a subsequent decrease in adults (Oliveira-Silva et al., 2007; Aujla and Huntley, 2014). The MMP-9 gene expression in the adult brain is maintained at a very low level, however, it rises significantly in certain conditions, e.g., during the increased plasticity/activity of neurons. It was also found that in pathological conditions abnormal MMP-9 release contributes to the development or influences the course of many brain disorders, including epilepsy, autism spectrum disorders, brain strokes, brain tumors, neurodegeneration, brain injuries, schizophrenia, and alcoholism (Reinhard et al., 2015; Vafadari et al., 2016; Pijet et al., 2018; Beroun et al., 2019; Bitanihirwe and Woo, 2020; Go et al., 2020; Yin et al., 2020; Gore et al., 2021). MMP-9 has been shown to mediate inflammation in nervous tissue in dementia (Panda and Soni, 2022). There is a lot of evidence that MMP-9 could represent a pathophysiological link between Alzheimer’s disease (AD) (Kaminari et al., 2018). Also, potential (transient) blood-brain barrier (BBB) dysfunction in neuropsychiatric systemic lupus erythematosus (NPSLE) may be caused by changes in MMP-9 serum levels (Deijns et al., 2020).

### 1.2. Functional polymorphism of MMP-9-1562C/T

The article describes the single nucleotide polymorphism of the MMP-9-1562C/T gene (rs3918242) and its links to brain disorders. The MMP-9-1562C/T polymorphism is characterized by the replacement of cytosine (C) with thymine (T) at the position 1562 bp in the MMP-9 gene promoter. Of course, the frequency of mutations varies among different population groups (Rodriguez-Murillo and Greenberg, 2008). What is particularly important, is this polymorphism has a functional effect due to its influence on MMP-9 expression levels. If the allele T is present, then the MMP-9 gene promoter activity is 1.5 times higher, when compared to the allele C. These findings result from analyses in the MALU macrophage cell line (Zhang et al., 1999). However, these relationships were not confirmed by studies conducted in primary cell cultures of amniotic epithelial cells, WISH (HeLa derivative), or THP-1 (human leukemia monocytic) cell lines, where no differences were found in the activity between the alleles T and C (Ferrand et al., 2002). To date, no studies have been conducted on the influence of this polymorphism on the MMP-9 promoter activity or the expression level in the brain cells or different types of cells. Recently, it was also demonstrated that carriers of the allele T show an increase in the grey matter volume (GMV), particularly pronounced in the right cerebral hemisphere, in the inferior parietal lobule (IPL), when compared to people with the allele C (Gregory et al., 2019). Additionally, this SNP was associated with changes in the gray matter volume bilaterally in the posterior part of the insula and the dorsolateral prefrontal cortex (DLPFC) in the left cerebral hemisphere (Gregory et al., 2019). Furthermore, carriers of the allele T had increased GMV, when compared to people with the allele C. Using functional magnetic resonance imaging it has been found that individuals with the allele T showed an increased brain activation associated with a working memory in the inferior parietal lobule when compared to subjects with the allele C (Gregory et al., 2019). However, the authors of the paper suggest that the MMP-9-1562C/T polymorphism association with schizophrenia risk is not related to the polymorphism-driven changes in MMP-9 expression, but rather with its genomic linkage with a nearby schizophrenia predisposing SLC12A5 gene, encoding KCC2 (a neuronal specific K + -Cl– cotransporter) (Gregory et al., 2019). To support this thesis they indicate that a relationship between the MMP-9-1562C/T polymorphism and the MMP-9 was only found in macrophages (Zhang et al., 1999; Rodriguez-Murillo and Greenberg, 2008). Later studies in primary cell cultures of amniotic epithelial cells, WISH or THP-1 cells, could not confirm the changes (Ferrand et al., 2002). Thus, there is no evidence that this polymorphism affects the activity of the MMP-9 promoter in the brain (Gregory et al., 2019). There is no other data that would show the effect of this polymorphism on the organism as a whole, on the organ system, or on the cells.

One of the hypotheses suggests that when the allele C is present, a nuclear repressor protein binds to the MMP-9 promoter, and this results in lower activity, and in turn, this protein is not able to bind the MMP-9 promoter in the presence of the allele T resulting in an increased activity of the promoter (Zhang et al., 1999; Nyati et al., 2010). So far, however, no one has investigated the molecular basis of the MMP-9-1562C/T polymorphism-dependent transcriptional regulation of the MMP-9 gene.

**FIGURE 1:**
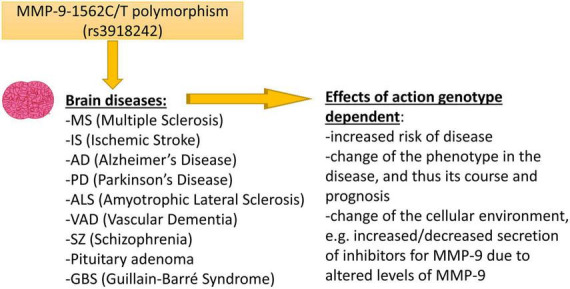
The figure shows brain diseases affected by the -1562C/T polymorphism in the MMP-9 gene and possible effects of action related to the occurrence of this polymorphism.

As we have mentioned earlier, the MMP-9-1562C/T polymorphism is localized in the gene promoter. It is known that when SNPs occur in transcription regulatory components, then it may affect the gene expression (Robert and Pelletier, 2018). The NCBI database shows that over one trillion of SNPs have been found in the human genome so far, of which only a small part is functionally important, and the MMP-9-1562C/T polymorphism should definitely be included among them. The MMP-9-1562C/T polymorphism may result in a change of the MMP-9 mRNA level, and can influence the possibility of an occurrence of diseases, as well as the course of the MMP-9 dependent brain disorders in humans (Figure 1). Therefore, it is not surprising that many publications were analyzing the existence of a relationship between MMP-9-1562C/T polymorphism and neurological disorders. Our publication aims at presenting all these efforts. However, it should be remembered that although there were publications in which, depending on the C or T allele in the MMP-9-1562C/T polymorphism, the expression level of MMP-9 changed (Glebauskiene et al., 2017). Then the protein level changed (Montaner et al., 2003) and finally the amount of MMP-9 in the serum changed (Fernandes et al., 2012; Valado et al., 2017; Li et al., 2018) the mechanism of action of this polymorphism from the molecular and functional side was not-thoroughly investigated. The fact that there are still publications showing the relationship of this polymorphism with various diseases shows that there is a lot of interest in this topic. However, we believe that many studies are still needed on the impact of this polymorphism on the activity and expression of the MMP-9 gene to show the molecular mechanism of its action.

### 1.3. The functional polymorphism of MMP-9-1562C/T in diseases not related to the brain

The discussed polymorphism was analyzed not only in the context of the central nervous system diseases Certain reports indicate its association with cardiovascular diseases (Mittal et al., 2014), prostate cancer (Kiani et al., 2020), gastric cancer (Peng et al., 2017), T-cell acute lymphoblastic leukemia (Lin et al., 2017), autoimmune diseases (Li et al., 2017), and chronic periodontitis (Mashhadiabbas et al., 2019). For example, the allele T results in the increase in MMP-9 levels in the serum of cardiovascular patients, and it is associated with a nearly doubled risk of cardiac death and significantly lower survival rates (Blankenberg et al., 2003). The genetic analysis of the MMP-9-1562C/T polymorphism showed a higher frequency of the allele T in patients with coronary heart disease, vs. the control group (Wu et al., 2013). It was also demonstrated that the increased MMP-9 levels in people with the allele T contributes to the exacerbation of the atherosclerosis and coronary artery stenoses (Spurthi et al., 2012). The recent meta-analysis showed that CT and TT genotypes were associated with the increased risk of coronary heart disease when compared to the CC genotype. This association was found in a group of Asians (among which the largest group were patients from China), but not in Europeans (Hassanzadeh-Makoui et al., 2020). The -1562C/T polymorphism is also significantly correlated with the frequency of myocardial infarction. The risk of myocardial infarction is higher in people with the CT or TT genotype than in people with the CC genotype (Juan et al., 2014). The –allele T was more frequently found in patients with acute myocardial infarction, vs. the control group. The frequency of the CT, and TT genotypes was higher in patients with morbidity and mortality caused by myocardial infarction than in people without such complications. The serum MMP-9 levels were significantly higher in patients with acute myocardial infarction, when compared to the control, and more strongly associated with the TT genotype (Abd El-Aziz and Mohamed, 2016). The MMP-9 -1562C/T polymorphism is also associated with the risk of development of hypertension (Yang et al., 2015). In the case of neoplasms, e.g., people with CT + TT genotype, who additionally had diabetes and smoked, were at a 3.52 times higher risk of developing prostate cancer (Kiani et al., 2020). In the case of gastric cancer, the meta-analysis showed that carriers of the TT genotype were at 1.666 times higher risk of that cancer when compared to carriers of the CC genotype (Peng et al., 2017). The most recent publications also report the relationship between the MMP-9-1562C/T polymorphism and the risk of pre-eclampsia or breast cancer and its aggressiveness (Gannoun et al., 2021; Bartnykaitė et al., 2022; Huang et al., 2022; Yan et al., 2022). It shows that the MMP-9-1562C/T polymorphism has a functional effect not only on brain cells but also on other cell types.

## 2. Brain disorders associated with the MMP-9-1562C/T polymorphism

### 2.1. Multiple sclerosis

Multiple sclerosis (MS) is a chronic disease of the CNS resulting in disability and is characterized by the presence of demyelinating the plaques in white matter of the brain. At the active stage of the disease, the blood-brain barrier (BBB) is damaged, resulting in the leakage of plasma proteins into the brain parenchyma. MMP-9 plays an important role both in the BBB degradation process and in demyelination. The polymorphism of MMP-9-1562C/T affects the activity of the MMP-9 promoter, may also affect the fluctuations of MMP-9 in multiple sclerosis. Therefore, the relationship between the MMP-9-1562C/T polymorphism and MS was investigated (Sabbagh et al., 2019).

Before proceeding with the meta-analyzes and research related to the influence of the single nucleotide polymorphism on brain disease pathology, it is worth approaching this subject from the genomic perspective. Genome-wide association study (GWAS) performed on 3,002 individuals (1,652 cases and 1,350 controls) of the German population, showed a nominally significant effect with a *p*-value of 0.02936, OR referring to the minor allele (T) = 0.82, CI = 0.69–0.98. This indicated that MS patients have a reduced frequency of the allele T in MMP-9-1562C/Tpolymorphism. A greater reduction of T-alleles in female MS patients was shown after conducting separate calculations for females and males (OR 1.26 vs. 1.16) (Sabbagh et al., 2019). This meta-analysis after the application of a dominant genetic model showed additionally no significant association between SNPs within the MMP-9 gene and MS susceptibility (OR = 1.12, *p* = 0.57451) (Sabbagh et al., 2019).

The earliest publication concerning the discussed relationship showed no correlation between the MMP-9-1562C/T polymorphism and a risk of MS occurrence in Swedish people. Genotype frequencies in Swedish MS patients were 143 for genotype CC and 56 for genotype CT + TT (absolute and relative frequency numbers are, respectively, 0.72 and 0.30) (Nelissen et al., 2000). Further studies in a group of people from Serbia demonstrated a reduced incidence of the allele T in MS female patients, vs. healthy women (χ2 = 6.12, df = 1, *p* = 0.01), and this suggested its protective action, in which the presence of the allele T reduces the risk of MS development in women. Allele T frequency in female patients was 10%, and in healthy females was 17% (Živković et al., 2007). These results were supported by studies by Benesová et al. (2008) conducted on the European population. They confirmed that the T allele carriers were less frequent in the female patients with MS-11.3% compared with 19.5% in healthy females—(Pa = 0.01, Pacorr = 0.05) (Benesová et al., 2008). Studies conducted in the Russian population also showed that the allele C of the MMP-9-1562C/T polymorphism is involved in MS development. They detected a significant link/association between MS and the allele C of MMP9–1562C/T polymorphism (χ2 = 4.1, *p* = 0.04) (Makarycheva et al., 2011).

Contrary to previous research, studies on the Polish population showed that the presence of the allele T was associated with an increased risk of MS development (*p* = 0.003; OR, 1.7). The CT + TT genotype was more often found in MS patients, regardless of their sex, when compared to healthy people (*p* = 0.0060). It was also identified that carriers of the genotype CC of the MMP-9-1562C/T polymorphism tended to be younger of at the onset of MS symptoms compared to carriers of the T allele (CT + TT genotype) (*p* = 0.0046) (Mirowska-Guzel et al., 2009). Studies conducted by the La Russa team in patients from Italy also showed that the allele T was significantly more frequently found in MS patients, vs. healthy people (*P* = 5.6 × 10^–5^; *P*adj = 4.0 × 10^–4^). The presence of the CT + TT genotype in MS patients and controls was, respectively, 79 and 26 compared to CC genotype where the numbers were 164 and 147, respectively (La Russa et al., 2010). The latest studies also showed that CT and CT + TT genotypes, and-the allele T increased the risk of MS in patients from Egypt. CT, CT + TT genotypes, and T allele carriers were found mostly among MS patients compared to healthy people (*p* = 0.009). In terms of the disease phenotype, MS patients with the allele T did not differ significantly from carriers with the allele C (Ibrahim et al., 2019). These findings were also confirmed in a group of patients from Iran. It was demonstrated that the allele T of the MMP-9-1562C/T polymorphism was associated with an increased risk of susceptibility to multiple sclerosis, therefore, it may predispose them to MS development. The T allele frequencies between MS patients compared to healthy volunteers were, respectively, 71.5 and 40% (*p* < 0.001) (Sabbagh et al., 2019).

The other studies showed that despite a lack of the MMP-9-1562C/T polymorphism influence on MS development, it has modified the course of this disease (Fernandes et al., 2009). Significant differences were observed when the genotype and allele frequencies of the MMP-9-1562/T polymorphisms were analyzed in MS patients subgrouped by clinical status (all *p* < 0.0001) (Fernandes et al., 2009). In the meta-analysis conducted to explain the existence of a relationship between the MMP-9-1562C/T polymorphism and MS incidences, covering 10 studies on MMP-9-1562C/T SNP with 1,757 MS patients and 1,702 control subjects, a strong positive correlation was shown between the discussed polymorphism and the risk of MS incidence. TT vs. CC model (OR = 3.20, 95% CI = 1.87–5.46, *p* < 0.001), and CT vs. CC model (OR = 1.53, 95% CI = 1.02–2.28, *p* = 0.04) increased the risk of MS susceptibility (Mohammadhosayni et al., 2020).

Furthermore, it was demonstrated that the MMP-9-1562C/T polymorphism was associated with varied plasma MMP-9 levels in MS patients. Patients with the CT + TT genotype had higher plasma MMP-9 levels (2.13 ± 0.016) vs. patients with the CC genotype (1.21 ± 0.06) (*p* < 0.0001) (Fernandes et al., 2012). That relationship was not confirmed by studies of Valado et al. (2017), but they found that in the control group (healthy subjects), the presence of the T allele was associated with higher plasma MMP-9 levels (*p* = 0.008). The latest study showed that plasma MMP-9 levels were higher in treatment-naive patients with relapsing–remitting multiple sclerosis (RRMS) when compared to healthy subjects (Samangooei et al., 2021). Furthermore, plasma MMP-9 levels in the kin of the first degree of MS patients (high risk subjects) were higher than in the control group, but this result was not statistically significant (*p* = 0.208) (Samangooei et al., 2021). Both in treatment-naive RRMS patients and in the kin of the first degree of MS patients (high risk subjects), the plasma MMP-9 level was higher in women (*p* = 0.011). A decrease in the plasma MMP-9 level with age was also observed, both in treatment-naive RRMS patients and in subjects from the high risk group (*p* = 0.001 and *p* = 0.025, respectively) (Samangooei et al., 2021). This research group strongly recommends the use of MMP-9 as a biomarker in MS pathogenesis (Samangooei et al., 2021).

In the case of the relationship between the MMP-9-1562C/T polymorphism and the risk of multiple sclerosis, it can be said that the T allele of the MMP-9-1562C/T polymorphism is associated with a higher incidence of multiple sclerosis, as well as with a worse course of the disease. There have been also studies in which it was described that the T allele, compared to the C allele, has a protective function and will prevent the development of MS, but they are entirely old and there are only a few of them. It can be also concluded that patients carrying the T allele in their genotype have higher serum levels of MMP-9 compared to patients with the C allele, which would suggest using of MMP-9 as a marker in the pathogenesis of MS.

### 2.2. Brain stroke

**FIGURE 2:**
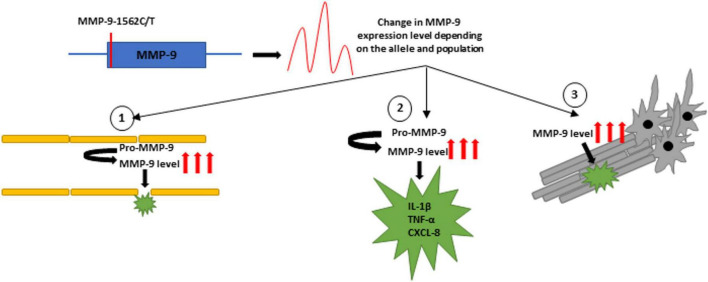
The figure shows possible mechanisms of the MMP-9-1562C/T polymorphism, which may affect the expression of the MMP-9 gene. Higher levels of MMP-9 in strokes may contribute to (1) BBB disruption, (2) activation of pro-inflammatory cytokines and chemokines, and (3) destruction of MBP.

Ischemic stroke (IS) is a sudden focal CNS injury of vascular origin. An increased level of MMP-9 expression was shown in the serum and brain tissues of IS patients (Nie et al., 2014; Abdelnaseer et al., 2017). It was also demonstrated that higher serum MMP-9 levels at the acute IS stage were associated with a higher risk of death and severe disability (Zhong et al., 2017). There are several mechanisms of how MMP-9 may affect the environment in strokes. The greatest danger is the effect of MMP-9 on the disruption of the blood-brain barrier (BBB) by digesting type IV collagen and occluding and claudin, essential components of tight junctions proteins (TJPs) in the BBB (Rosell et al., 2008; Yamada et al., 2013). MMP-9 is also involved in the immune response during strokes. Factors such as TNF-α and IL-6 activate the expression of MMP-9, and metalloprotease, in turn, is involved in the subsequent activation of IL-1β and CXCL-8 (Zhu et al., 2022). In addition, MMPs are capable of proteolytic degrading of myelin basic proteins (MBP) (Chandler et al., 1995; Figure 2).

**FIGURE 3:**
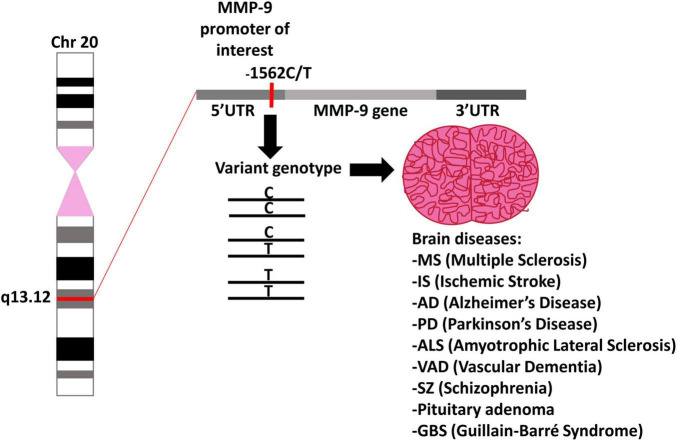
MMP-9 gene polymorphism. A schematic representation of chromosome 20 with the location of the MMP-9 gene as well as possible genotypes of the MMP-9 gene polymorphism and their impact on brain related diseases.

As we mentioned earlier expression of MMP-9 has changed and higher serum level was connected with a higher risk of death in IS patients. This suggests that serum MMP-9 levels may be an important prognostic factor in IS. The MMP-9-1562CT polymorphism affecting the promoter of the MMP-9 gene may also cause a change in the expression of the MMP-9 gene and, consequently, a change in the level of MMP-9 in the plasma. Therefore numerous studies were conducted, focusing on a relationship between the MMP-9-1562C/T polymorphism and brain strokes.

Studies conducted in the Polish population did not show a relationship between the -1562C/T polymorphism in the MMP-9 gene and the risk of ischemic stroke of varying etiologies. They studied 222 patients with cardioembolic stroke (CE), 100 stroke patients with small vessel disease (SVD), and 96 stroke patients with large vessel disease (LVD). The control group was composed of 408 subjects. The CC, CT, and TT genotypes of the MMP-9 gene were in Hardy–Weinberg equilibrium for patients with different stroke etiologies and for the total and control groups separately (*p* > 0.1 for each group) (Szczudlik and Borratyńska, 2010). Other studies in the Polish population demonstrated a higher occurrence of the T allele and the CT + TT genotype in patients with a stroke (90% of patients had the ischemic stroke) when compared to healthy subjects (OR 1.73, 95% CI 1.34–2.23 and 1.89, 95% CI 1.39–2.56, respectively). The patients with the T allele (CT + TT) were younger when the stroke occurred (63.5 ± 11.7 years), vs. patients with the CC genotype (71 ± 14.1 years) (*p* = 0.0002) (Buraczynska et al., 2015). A recent study of a 100 Indian patients and a 100 controls showed a similar association of the CT genotype (adjusted odds ratio [aOR] = 7.09; *P* < 0.001), TT genotype (aOR = 19.75; *P* < 0.001), and T allele (aOR = 10.71; *P* < 0.001) with a significant risk of ischemic stroke. The risk for the development of ischemic stroke in the Indian population was 10.71-times higher when the allele T occurs vs. allele C. This group of researchers also revealed that methylation in the MMP-9 gene promoter decreased the risk of stroke (aOR = 0.23; *P* < 0.001) (Choudhari et al., 2021). Montaner et al. (2003) conducted a study in the Spanish population and did not find any differences in the frequency of alleles of the -1562C/T polymorphism in the MMP-9 gene between healthy subjects and patients with acute brain stroke (CC/CT/TT: 79.7/20.3/0% vs. 72.3/27.7/0% respectively; *p* = 0.37). This group of researchers also showed that the plasma MMP-9 level was significantly higher in patients with acute brain stroke, who later had large parenchymal hemorrhages (PH) when compared to patients with acute brain stroke who did not have such hemorrhage (PH, 191.4 ng/mL; non-PH, 68.05 ng/mL; *p* = 0.022). The MMP-9 protein levels were slightly higher in patients with acute brain stroke who had the CC genotype when compared to the patients with the CT/TT genotypes (CC, 127.12 ng/mL; CT/TT, 46.31 ng/mL; *p* = 0.11) (Montaner et al., 2003).

Studies in the Chinese population showed that the TT genotype and presence of the allele T were associated with the increased risk of IS symptoms development, vs. the CC genotype. The ischemic stroke occurrence was 1.5 times higher in carriers of the allele T (OR = 1.543, 95% CI = 1.144–2.080, *p* = 0.004) (Nie et al., 2014). Another study was also conducted in that population, which showed that patients with the CC genotype and the CT + CC genotypes were at a significantly higher risk of ischemic stroke, vs. the TT genotype [ORs (95% CI) was 5.47 (2.64–12.38) and 1.55 (1.08–2.24), respectively]. Genotype frequencies in Chinese IS patients were 171 for genotype CC, 146 for genotype CT + TT and 39 for genotype TT. Furthermore, it also showed a positive relationship between the TC + CC genotypes of the -1562C/T polymorphism accompanied by smoking, and a risk of IS development OR (95% CI) was 2.03 (1.11–3.74) (Hao et al., 2015). Another study in the Chinese population confirmed that the CC genotype of the MMP-9-1562C/T polymorphism was associated with a higher risk of the ischemic stroke, when compared to the TT genotype [OR (95%CI) = 5.47 (2.64–12.38)]. Furthermore, a correlation between the polymorphism and the risk of the ischemic stroke development was analyzed, taking into account, among others, patients’ BMI. The TC + CC genotypes were associated with the increased risk of ischemic stroke in patients with higher BMI [OR (95%CI) = 1.81 (1.03–3.22)] (Zhao et al., 2015). Detailed studies were also conducted in a relatively large population of people from southern China, where a significant relationship between allele frequencies of the MMP9-1562C/T polymorphism between 1,274 IS patients and 1,258 controls was observed (*p* = 0.012 for the genotype and *p* = 0.0092 for the allele) (Li et al., 2018). The presence of the allele T of the -1562C/T polymorphism in the MMP-9 gene was higher compared to controls (OR = 1.32, 95% CI: 1.11–1.59, *p* = 0.0092) and was associated with a higher risk of IS development. Furthermore, the allele T was associated with a higher risk of large artery atherosclerosis (LAA) IS (*p* = 0.017). The serum MMP-9 level was significantly higher in IS patients with the CT and TT genotypes of the MMP-9-1562C/T polymorphism than in carriers of the main CC genotype (*p* = 0.031). Furthermore, patients with the genotype variants (CT + TT) of the MMP-9 polymorphism had larger stroke lesion volumes than carriers of the CC genotype (*p* = 0.036). The studies also showed that smokers being carriers of the allele T, which is associated with elevated MMP-9 levels, were at a higher risk of IS development (*p* = 0.022) (Li et al., 2018).

Many meta-analyses were conducted concerning a relationship between the -1562C/T polymorphism and IS development. The first meta-analyses showed that this polymorphism is not a risk factor for IS. Of the studies included in this meta-analysis, five concerned the ischemic stroke, one the hemorrhagic stroke, and one concerned with both ischemic and hemorrhagic stroke. No significant association was detected in the T allele vs. C allele [OR 0.98, 95% CI (0.84, 1.15), *p* = 0.84], nor in the dominant genetic model [OR 0.95, 95% CI (0.81, 1.13), *p* = 0.59], the recessive genetic model [OR 1.55, 95% CI (0.86, 2.81), *p* = 0.15] (Fan et al., 2014). The second meta-analysis also did not show a relationship between the MMP-9-1562C/T polymorphism and a risk for the ischemic stroke development. The overall ORs and 95% CIs of MMP-9 -1562T were 0.78, 0.59–1.02 (*p* = 0.460) and 1.65, 0.73–3.75 (*p* = 0.340) compared with C in the dominant and recessive models, respectively (Wen et al., 2014). However, the fact that the quantity of data available at that time was lower than the quantity used in later meta-analyses should also be considered. The first meta-analysis was based on seven studies of 3,149 patients and the second on three studies and 1,087 patients (Fan et al., 2014; Wen et al., 2014). Meta-analyses conducted later showed a significant relationship between the MMP-9-1562C/T polymorphism and a risk of ischemic stroke. All three late meta-analyses showed that in the Chinese population, the allele T and the TT + CT genotype were associated with the increased likelihood of ischemic stroke, vs. the allele C and the CC genotype (He et al., 2017; Misra et al., 2018; Wang et al., 2018). Furthermore, two of those meta-analyses showed that this polymorphism had functional importance only in the Chinese population, but there was no relationship between its occurrence and the risk of ischemic stroke in the European group (He et al., 2017; Wang et al., 2018). The meta-analysis published by He et al. (2017) included 3 studies performed on 801 cases and 877 controls in the European population, and 11 studies on 2,432 cases and 2,246 controls in the Chinese population. In the Chinese population T vs. C was OR = 1.60, 95% CI = 1.41–1.82, *p* < 0.001; TT + CT vs. CC: OR = 1.48, 95% CI = 1.28–1.71, *p* < 0.001 and in the European population T vs. C was OR = 1.30, 95% CI = 0.79–2.13, *p* < = 0.305; TT + CT vs. CC: OR = 1.30, 95% CI = 0.68–2.49, *p* = 0.423 (He et al., 2017). Wang et al. analyzed sixteen original studies including 3,647 stroke patients and 3,685 unrelated controls. Overall analysis showed that MMP-9 gene rs3918242 polymorphism significantly increases stroke risk (T vs. C; TT vs. CC; CT vs. C; TT + CT vs. CC; TT vs. CT + CC; all *p* < 0.05). However, stratification by ethnicity showed a significant association between MMP-9-1562C/T polymorphism and increased stroke risk only among Asians, but not among Europeans (TT + CT vs. CC) (Wang et al., 2018). However, one of those meta-analyses showed a significant relationship between the recessive model of the MMP-9-1562C/T polymorphism (TT vs. CC + CT) and the risk of ischemic stroke in the European population (OR 2.06; 95% CI 1.14–3.73; *p*-value = 0.02) (Misra et al., 2018). The last meta-analysis from 2020 showed a relationship between the -1562C/T polymorphism in the MMP-9 gene and the increased risk of IS incidence in Asians: the T vs. C comparison gave an OR of 1.419 with a 95% CI of 1.244–1.620; (TT vs. CC) gave an OR of 2.113 with a 95% CI of 1.339–3.332; and the (TT vs. CT + CC) analysis indicated an OR of 1.996 with a 95% CI of 1.259–3.164. Also, a significant association was found in the European population: (TT vs. CC): 2.708 (95% CI, 1.417–5.173); (TT vs. CT + CC): 2.487 (95% CI, 1.308–4.732). Additionally significant associations were found between MMP-9-1562C/T polymorphism and risk for IS, both in men and women, as well as in people below and above 65 years of age, with or without diabetes, and smoking. 5,630 cases and 5,368 controls from nineteen studies (16 were of Asians, whereas the other 3 were of whites) were included in this study (Wu et al., 2020).

Furthermore, the MMP-9-1562C/T polymorphism was also studied in a context of early neurological deterioration (END) in brain strokes. Early neurological deterioration is relatively common in patients with acute ischemic brain stroke and is associated with an increased mortality rate (Yi et al., 2019a). In the paper by Yi et al. (2019a), it was demonstrated that the CT/TT genotype of MMP-9-1562C/T was significantly statistically associated with a moderate or severe stroke. Among patients with moderate or severe strokes, the frequency of CT/TT was significantly higher than in patients who are carriers of wild CC type (31.8% [35/110] vs. 15.2% [77/505], respectively, *p* < 0.001). It was also demonstrated that the END frequency was higher in patients with the CT/TT genotype, vs. patients with the CC genotype (30.0% [33/110] vs. 14.9% [75/505], respectively, *p* < 0.001). This prospective study proposed a possible role of the MMP-9 gene polymorphism in higher END risk in patients with atrial fibrillation (Yi et al., 2019a). Generalized multifactor dimensionality reduction (GMDR) analysis showed a gene-gene interaction among tumor protein P53 rs1042522 C > G, mouse double minute 2 homolog MDM-2 rs2279744 T > G, and MMP-9-1562C/T. Interaction between these three variants was connected with a higher risk of neurological deterioration and poor functional outcome after IS (genotypes CC vs. GG; TT vs. GG; CC vs. TT, respectively, gave an OR of 2.74 with 95% CI 1,31–5,96, *p* = 0.003) (Yi et al., 2019b).

Generally, the T allele and the CT + TT genotypes appear to be responsible for the increased risk of strokes. Most studies, as well as meta-analyses, indicate the association of the presence of the T allele of the MMP-9-1562C/T polymorphism in patients with a higher risk of stroke, as well as with early neurological deterioration and higher mortality compared to allele C carriers.

### 2.3. Neurodegenerative diseases

MMP-9, a major component of the vascular basement membrane of arteries, may contribute to the pathogenesis of neurodegenerative diseases such as Alzheimer’s disease (AD), Parkinson’s disease (PD), and amyotrophic lateral sclerosis (ALS) (He et al., 2013). MMP-9 causes neuronal death mediated by neurotoxins through the NF-κB induction and AP-1 binding to its gene promoter (Kim et al., 2010). The MMP-9 proenzyme level is elevated in the plasma of AD patients (Baig et al., 2008), what could be related to MMP-9-1562C/T polymorphism. Accordingly, an expression of the tissue inhibitors of MMPs is elevated in the cerebrospinal fluid of PD patients, and the skin, serum, and cerebrospinal fluid of ALS subjects (He et al., 2013).

Genome-wide association study meta-analysis conducted mainly on participants of European origin showed a little evidence that MMP-9 expression quantitative trait loci (eQTLs) and protein quantitative trait loci (pQTLs) affected the odds of Alzheimer’s disease (*p* = 0.45 and *p* = 0.51) (Anderson et al., 2022).

In 2003, Helbeque et al. demonstrated that the MMP-9-1562C/T polymorphism is associated with the reduced risk for dementia development in the course of Alzheimer’s disease in the French population. Patients without the apolipoprotein E4 allele (APOE e4), being a factor contributing to AD development, but with the allele T of the -1562C/T polymorphism in the MMP-9 gene were at a lower risk of dementia development when compared to carriers of the allele C [OR = 0.6 (95% CI, 0.3–1.0), *p* = 0.05] (Helbecque et al., 2003). Similarly, Flex et al. (2006) showed that MMP-9-1562C/T polymorphism, or rather a genotype TT is positively correlated with the development of vascular dementia in the Italian population (OR = 6.8, 95% CI, 1.3–35.1, *p* = 0.02). The TT genotype was significantly more common in VAD patients vs. the control (*p* = 0.03). However, the frequency of the TT, CT, and CC genotypes did not differ significantly between the patients with Alzheimer’s disease and the control subjects (TT vs. CC: OR = 4.8, 95% CI, 0.9–23.8, *p* = 0.052; TT vs. CT: OR = 4.4, 95% CI, 0.7–25.7, *p* = 0.098; CT vs. CC: OR = 1.0, 95% CI, 0.6–1.7, *p* = 0.819) (Flex et al., 2006). However, a few years later, the same research group demonstrated in the Italian population that the frequency of the TT genotype in AD patients was significantly higher when compared to healthy people (1.4%; *p* = 0.027) (Flex et al., 2014). AD patients were twice more frequently the carriers of the TT genotype of the -1562C/T polymorphism in the MMP-9 gene when compared to the control group (Flex et al., 2014). Other studies, using tissues collected from AD patients from England, showed that there was no relationship with the MMP-9-1562C/T polymorphism (Baig et al., 2008). In that case, the frequency of the CC and CT/TT genotype did not differ significantly between controls and AD subjects (*p* = 0.413 and *p* = 0.367), and was also not associated with APOE e4 (control *p* = 1.0, AD *p* = 0.799) (Baig et al., 2008).

In studies conducted in the Chinese population, a relationship between the MMP-9-1562C/T gene polymorphism and a risk of PD or ALS development were analyzed, and it was found that it is a risk factor for those two diseases. There were significant differences between sick patients and control individuals in allele frequencies C vs. T (for PD: *p* < 0.001; for ALS: *p* = 0.002) and in genotype CC vs. CT + TT frequencies (for PD: *p* < 0.001; for ALS: *p* = 0.006) (He et al., 2013).

In conclusion, in the case of neurodegenerative diseases, it is difficult to determine which allele or genotype in the MMP-9-1562C/T polymorphism is associated with a higher probability of developing the diseases. There is insufficient literature data on this subject and it requires further research.

### 2.4. Schizophrenia

MMP-9 influences the function of the hippocampus and the prefrontal cortex, and as a consequence it is suspected of being involved in the development of schizophrenia (SZ), in which damage to the prefrontal cortex is one of the most common pathological symptoms (Ali et al., 2017). In the clinical studies, an increased MMP-9 expression was observed in samples of blood from schizophrenic patients (Devanarayanan et al., 2016; Ali et al., 2017). Furthermore, abnormal peripheral expression and disrupted methylation of the MMP-9 gene were demonstrated in SZ patients (Gao et al., 2018). Changes in the expression of the MMP-9 gene may be dependent on the MMP-9-1562C/T polymorphism.

In the first studies of the Polish population on a relationship between the -1562C/T polymorphism and schizophrenia, a much higher occurrence of the CC genotype was found compared to CT + TT genotypes in the schizophrenic patients than in the healthy population (*p* = 0.032 OR = 1.39 95% CI 1.03–1.89). The study was carried out in a group of 442 schizophrenic patients and 558 healthy control subjects (Rybakowski et al., 2009). However, two subsequent studies, also conducted on the Polish population, did not confirm these results. Groszewska et al. in a study conducted on 147 patients with SZ did not find a statistically significant relationship between the analyzed polymorphism and schizophrenia. There were no statistically significant differences in the transmission of individual alleles of the MMP-9-1562C/T polymorphism (*p* = 0.241) (Groszewska et al., 2011). Also, Bienkowski et al. did not find any differences in the frequency of genotypes and alleles of the MMP-9-1562C/T polymorphism between SZ patients and the control group in the Polish population (*p* = 0.3 and *p* = 0.1, respectively). Furthermore, no relationship was found between this functional polymorphism and different (deficit and non-deficit) SZ groups. No differences were found in genotype (*p* = 0.2) and allele frequencies (*p* = 0.08) between non-deficit patients and the controls and there were no differences in genotype (*p* = 0.6) and allele frequencies (*p* = 0.4) between the deficit patients and the controls (Bienkowski et al., 2015). In the Egyptian population, the occurrence of the genotypes of the MMP-9-1562C/T polymorphism did also not differ significantly between the control and the SZ patients (*p* = 0.396). The research was conducted on 44 SZ patients and 50 healthy subjects. However, this research group discovered that patients who are carriers of the CC genotype in the -1562C/T polymorphism of the MMP gene scored higher on the Positive and Negative Syndrome Scale (PANSS) in SZ than subjects with the CT/TT genotype (*p* = 0.007), and this may imply a possible relationship between MMP-9-1562C/T SNP and exacerbation of the clinical manifestation of schizophrenia (Ali et al., 2017). A study in the Chinese population (performed on 298 patients with schizophrenia and 298 controls) showed that the T allele of the MMP-9-1562C/T polymorphism of the MMP-9 gene may predispose an occurrence to SZ, and this risk is 1.6 times higher (C allele vs. T allele: odds ratio = 1.564, 95% CI: 1.005∼2.436, *p* = 0.046) (Han et al., 2011). Furthermore, a relationship between the discussed polymorphism and schizophrenia in patients infected with *Toxoplasma gondii* was demonstrated in the Lebanese population. It was found that all schizophrenic patients who did not have antibodies against *Toxoplasma gondii* had the wild type CC genotype (*p* = 0.003), while patients diagnosed with schizophrenia and with the positive result for the antibodies against *Toxoplasma gondii*, possessed the allele T of MMP-9-1562C/T polymorphism. The frequencies of muted allele T was significantly higher in patients with toxoplasmosis compared to those who have not had the infection (67.5, 74.0%; *p* < 0.001) (El Mouhawess et al., 2020).

In the case of schizophrenia, we think that it is also impossible to determine which allele could be unambiguously responsible for the development of the disease. Some studies indicate that the C allele and the CC genotype, compared to the T allele and the CT + TT genotype, seem to be responsible for a higher risk of schizophrenia, as well as worsening of the symptoms. However, there have also been studies indicating the T allele may be connected with an increased incidence of SZ. In the case of this disease, more research is needed to examine the relationship between the MMP-9-1562C/T polymorphism and the risk of developing schizophrenia.

### 2.5. Brain tumors

It was demonstrated that in tissues of glioblastoma multiforme, the MMP-9 gene expression is elevated and increases along with the tumor grading (Komatsu et al., 2004). This mechanism may be related to the presence of the MMP-9-1562C/T polymorphism, which affects the activity of the MMP-9 promoter (Zhang et al., 1999). An analysis of the correlation between the MMP-9-1562C/T polymorphism and the risk of incidence of all 4 astrocytoma grades according to the WHO scheme conducted in the Chinese population, did not find this association. MMP-9 genotypes and allelotypes did not differ significantly between the astrocytoma patients and the control group (0.818) (Lu et al., 2007).

Studies in patients with invasive pituitary adenomas showed a significantly higher MMP-9 expression when compared to non-invasive pituitary adenomas (Gong et al., 2008). However, Glebauskiene et al. (2017) demonstrated that in pituitary adenomas, the CC genotype of the -1562C/T polymorphism of the MMP-9 gene was correlated with lower expression of the MMP-9 gene, but differences were not statistically significant (*p* = 0.9). Genotype CC was also more frequent in a group of patients with this neoplasm when compared to healthy people (81.4 vs. 64.6%, *p* = 0.002). The CC genotype was more frequently observed in non-recurring, inactive pituitary, non-invasive, and invasive adenomas when compared to the control (81.0% vs. 64.6%, *p* = 0.041; 81.8% vs. 64.6%, *p* = 0.005; 100.0% vs. 64.6%, *p* < 0.001; 81.8% vs. 64.6%, *p* = 0.021; respectively) (Glebauskiene et al., 2017).

Similarly, in the case of the association of the MMP-9-1562C/T polymorphism with an increased risk of brain tumors, there are not enough studies to draw any conclusions.

### 2.6. Guillain–Barré syndrome

Guillain-Barré syndrome (GBS) is a progressive disease of the peripheral nervous system with an immunological background (Hayat et al., 2020). Matrix metalloproteinase 9 is an inflammatory mediator regulating the composition of the extracellular matrix through degradation of its components, such as elastins, collagens, and proteoglycans (Donohoe et al., 1999). In GBS patients in the progressive phase of the disease, elevated levels of pro-inflammatory cytokines, such as TNF-a and IL-1b, and metalloproteinase MMP-9 are found (Hayat et al., 2020). In patients with Guillain-Barré syndrome, the expression of MMP-9 and pro-inflammatory cytokines decreases in the recovery stage (Hayat et al., 2020). The MMP-9-1562C/T polymorphism affects the activity of the MMP-9 promoter (Zhang et al., 1999) and is presumed to also affect the expression of the MMP-9 gene. Differences in the expression of the MMP-9 gene may also affect the change in the overall intracellular environment, and thus also the activity of pro-inflammatory cytokines. It is indicative that MMP-9 contributes to the GBS pathogenesis.

A study conducted on 263 patients of the European race showed that -1562C/T SNP in the MMP9 gene was significantly correlated with the disease exacerbation in GBS patients (*p* = 0.01) but no relationship between this polymorphism and higher GBS incidence was observed (Geleijns et al., 2007). Furthermore, it was demonstrated that the polymorphic variant of the SNP allele in the MMP-9-1562C/T gene was more frequent in patients with severe GBS, when compared to patients with mild GBS (13.6 vs. 5.7%, OR = 2.6, 95% CI = 1.1–6.0, *p* = 0.02) (Geleijns et al., 2007). These results were confirmed by Hayat et al. in studies in the Bangladesh population (303 patients with GBS and 303 healthy controls). They demonstrated that the genotype and the frequency of alleles of the MMP-9-1562C/T polymorphism does not differ significantly between the GBS patients and the healthy subjects (*p* = 0.665 and *p* = 0.479, respectively). Furthermore, no significant relationship was found between this polymorphism and the GBS frequency (*p* = 0.47 for genotypes and *p* = 0.48 for alleles). However, a relationship between the increased frequency of the CT genotype and the T allele in patients with the severe GBS was found, when compared to a mild form of this disease (*p* = 0.01, OR = 2.28, 95% CI = 1.22–4.22; pc = 0.03 and *p* = 0.012, OR = 2.0, 95% CI = 1.14–3.38; pc = 0.024, respectively). Furthermore, the TT genotype dominated in GBS patients with a poor prognosis, when compared with patients with a good prognosis after 6 months from the onset of GBS (4.2 vs. 1.4%), but this relationship was not statistically significant. It was also demonstrated that the serum MMP-9 levels were significantly elevated in GBS patients when compared to healthy subjects, and in patients with axonal and demyelinating GBS, when compared to healthy subjects (*p* ≤ 0.0001 and *p* ≤ 0.0001, respectively). These authors suggest the role of MMP-9-1562C/T polymorphism in the severity of the GBS disease (Hayat et al., 2020). Concluding, it was demonstrated that the MMP-9-1562C/T polymorphism may predispose people to the development of severe GBS forms.

Data on the association of the MMP-9-1562C/T polymorphism with GBS indicate that this polymorphism is related to the course of this disease.

## 3. Conclusion

Metalloproteinase-9 (MMP-9) plays an important function in the physiology and pathology of the nervous system. The -1562C/T polymorphism in the MMP-9 gene influences the activity of its promoter, resulting in the modified expression of that metalloproteinase in the brain. In consequence, the MMP-9-1562C/T polymorphism influences the likelihood of development and the course of many brain diseases associated with abnormal MMP-9 expression found by many researchers (Table 1). The differences in frequencies of the genotypes and alleles of the MMP-9-1562C/T polymorphism observed in the discussed studies, as well as their relationship (or lack of it), with numerous CNS disorders, may result from genetic differences occurring between populations. As well as the presence of varied environmental factors to which individual populations are exposed, the number of research studies are significantly limited. It should also be noted that single nucleotide polymorphisms occur simultaneously in other genes in the body, and they may influence a risk of development of a given disease or modify its course, and the effects of these polymorphisms may overlap leading to diverse phenotypic results. Additionally, a stratification of a population, incorrect classification and statistical methods, as well as an insufficient number of subjects may be a cause of contradictory or ambiguous study results and controversies associated with the role of the MMP-9-1562C/T polymorphism in the risk of development and the influence on the course of CNS disorders. Definitively, to explain this role thoroughly, further numerous studies for this problem are required. Nevertheless, analyses presented in the publication clearly show a broad pathological role of the MMP-9-1562C/T polymorphism in the human brain disorders. Considering presented statistical data here and frequently conflicting results, it can be said that MMP-9-1562C/T polymorphism has brain disease risk and course modifying potential, but it is not one of the major pathological factors involved in brain disease development. However, the statement that the functional MMP-9-1562C/T polymorphism influences the course of many important neuropsychiatric diseases appears to be based on solid foundations, and at the same time suggesting an important pathological role of metalloproteinase MMP-9 in pathologies of the central nervous system.

**TABLE 1:** The table contains alleles and genotypes of the -1562C/T polymorphism of the MMP-9 gene, diseases that are affected by specific alleles and genotypes, populations in which the studies were conducted, appropriate references, number of cases and controls, *p*-value.

The allele/Variant	Disease	Study population	References	Cases and controls	*P*-value
Significant decrease of allele T in female patients with MS compared to healthy female controls	Multiple sclerosis	Serbian population	Živković et al., 2007	187 cases and 282 controls	*p* = 0.01
T allele less frequent in female MS patients	Multiple sclerosis	European population	Benesová et al., 2008	244 cases, 132 controls	*p* = 0.01
Association of allele C with MS	Multiple sclerosis	Russian population	Makarycheva et al., 2011	104 nuclear families, each of them comprising MS patients and their healthy parents	*p* = 0.04
Allele T/genotypes CT + TT associated with increased risk of MS	Multiple sclerosis	Polish population	Mirowska-Guzel et al., 2009	234 cases, 190 controls	*p* = 0.003/ *p* < 0.00001
Significant increase of T alleles in MS patients	Multiple sclerosis	Italian population	La Russa et al., 2010	243 cases, 173 controls	*p* = 5.6 × 10^–5^
Significant increase of allele T/genotypes CT, CT + TT in MS patients	Multiple sclerosis	Egyptian population	Ibrahim et al., 2019	50 cases, 100 controls	*p* = 0.009
Significant increase of allele T in MS patients	Multiple sclerosis	Iranian population	Sabbagh et al., 2019	100 cases, and 105 controls	*p* < 0.001
Significant increase of allele T/genotypes CT + TT in IS patients	Ischemic stroke	Polish population	Buraczynska et al., 2015	320 cases, 400 controls	*p* < 0.0001/ *p* < 0.0001
Significant increase of allele T/in IS patients	Ischemic stroke	Chinese population	Nie et al., 2014	396 cases, 400 controls	*p* = 0.004
Association of genotypes CC, CT + CC with increased risk of IS	Ischemic stroke	Chinese population	Hao et al., 2015	317 cases and 317 controls	*p* < 0.001/ *p* = 0.01
Association of genotype CC with increased risk of IS	Ischemic stroke	Chinese population	Zhao et al., 2015	335 cases, 335 controls	*p* < 0.001
Significant increase of allele T in IS patients	Ischemic stroke	Chinese population	Li et al., 2018	1274 cases, 1,258 controls	*p* = 0.0092
Association of allele T/genotypes CT + TT with increased risk of IS	Ischemic stroke	Chinese population	He et al., 2017	3233 cases, 3,123 controls	*p* < 0.001/ *p* < 0.001
Association of allele T/Genotypes CT + TT with increased risk of IS	Ischemic stroke	Chinese population	Wang et al., 2018	3647 cases, 3,685 controls	all *p* < 0.05
Association of genotype TT with increased risk of IS	Ischemic stroke	European population	Misra et al., 2018	923 cases, 1,016 controls	*p* = 0.02
Association of genotype TT with increased risk of IS	Ischemic stroke	Mixed population	Wu et al., 2020	630 cases, 5,368 controls	*p* = 0.005
Significant association of genotype CT/TT in patients with severe to moderate stroke	Severe to moderate ischemic stroke	Chinese population	Yi et al., 2019a	Severe IS-112 patients Moderate IS-503 patients	*p* < 0.001
Significant association of genotype CT/TT in patients with END	Early neurologic deterioration			Patients with END-108 Patients without END-507	*p* < 0.001
Association of allel T with a lower risk of developing dementia	Dementia in the course of Alzheimer’s disease	French population	Helbecque et al., 2003	229 cases, 253 controls	*p* = 0.05
Association of genotype TT with increased risk of VAD	Vascular dementia	Italian population	Flex et al., 2006	193 cases, 223 controls	*p* = 0.02
Significant increase of genotype TT in AD patients	Alzheimer’s disease	Italian population	Flex et al., 2014	533 cases, 713 controls	*p* = 0.027
Significant increase of allele C and genotype CC in SZ patients	Schizophrenia	Polish population	Rybakowski et al., 2009	432 cases, 558 controls	*p* = 0.032
Association of genotype CC with higher PANSS	Clinical manifestation of schizophrenia	Egyptian population	Ali et al., 2017	44 cases, 50 controls	*p* = 0.034
Association of allele T with increased risk of SZ	Schizophrenia	Chinese population	Han et al., 2011	298 cases, 298 controls	*p* = 0.046
Association of genotype CC with risk of pituitary adenoma	Pituitary adenoma	Lithuanian population	Glebauskiene et al., 2017	86 cases, 526 controls	*p* = 0.021
Significant increase of allele T and genotype CT with a severe form of GBS	Severe course of Guillain-Barré syndrome	Bangladesh population	Hayat et al., 2020	303 cases, 303 patients	*p* = 0.01

## Author contributions

SP-J: conceptualization and writing—original draft preparation. MR: supervision. Both authors: writing—review and editing, read, and approved the final manuscript.
